# Characteristics and Clinical Implications of Cytomegalovirus Infection in Patients with Drug-Resistant Ulcerative Colitis Undergoing Colectomy—Data from a Tertiary Referral Center in Poland

**DOI:** 10.3390/jcm14144823

**Published:** 2025-07-08

**Authors:** Estera Banasik, Paweł Kosikowski, Izabela Miechowicz, Piotr Zelga, Tomasz Banasiewicz, Agnieszka Dobrowolska, Piotr Eder

**Affiliations:** 1 Department of Gastroenterology, Dietetics and Internal Medicine, Poznan University of Medical Sciences, University Clinical Hospital, 60-355 Poznan, Poland; esta717@gmail.com (E.B.); agdob@ump.edu.pl (A.D.); 2 Department of Clinical Pathology, Poznan University of Medical Sciences, 60-355 Poznan, Poland; kosikowski.pawel@gmail.com; 3 Department of Computer Science and Statistics, Poznan University of Medical Sciences, 60-806 Poznan, Poland; iza@ump.edu.pl; 4 Department of General and Transplant Surgery, Poznan University of Medical Sciences, 60-355 Poznan, Poland; piotr.zelga@usk.poznan.pl; 5 Department of General and Endocrine Surgery and Gastroenterological Oncology, Poznan University of Medical Sciences, University Clinical Hospital, 60-355 Poznan, Poland; tbanasiewicz@op.pl

**Keywords:** colectomy, cytomegalovirus, immunohistochemistry, ulcerative colitis

## Abstract

**Background/Objectives**: This study aimed to assess the frequency, risk factors, and clinical implications of cytomegalovirus (CMV) colitis in patients undergoing colectomy due to refractory ulcerative colitis (UC). **Methods**: A retrospective analysis was conducted on patients with drug-resistant UC who underwent colectomy at a tertiary referral center between 2009 and 2017. Histological inflammatory activity in surgical specimens was assessed using the Simplified Geboes Score. The presence and density of CMV expression were estimated immunohistochemically. Preoperative clinical, biochemical, and endoscopic data, as well as the short- and long-term postoperative disease courses, were evaluated in relation to the presence of CMV colitis at the time of surgery. **Results**: CMV colitis was identified in 14% (7/49) of patients. The CMV-positive group exhibited significantly shorter disease durations and higher C-reactive protein concentrations at the time of surgery. This subgroup also demonstrated consistently numerically higher steroid use, both in terms of the usage frequency and cumulative treatment duration. Patients with concomitant CMV colitis had lower likelihoods of stoma closure and restoration of gastrointestinal continuity in the long-term. **Conclusions**: Concomitant CMV colitis is not uncommon in patients with treatment-refractory UC. Testing for CMV should be considered, particularly in individuals with a short-term, dynamic, and aggressive disease course unresponsive to standard therapy, especially steroids.

## 1. Introduction

Ulcerative colitis (UC), classified under the group of inflammatory bowel diseases (IBD), is a chronic condition of unknown etiology that affects the large intestine [1,2,3]. It predominantly impacts young individuals and may be associated with severe complications. Immunological disturbances observed in UC, along with the immunosuppressive treatments used for its management, significantly increase the risk of opportunistic infections [4]. Among these, cytomegalovirus (CMV) colitis is one of the least understood co-infections, with potentially serious clinical implications [5,6,7].

CMV, a member of the DNA beta-Herpesviridae family, is transmitted through prolonged contact with bodily fluids such as saliva, blood, semen, urine, and tears [8,9,10,11]. It is estimated that more than half of adults are infected with CMV. However, in most cases, the infection remains asymptomatic, with the virus persisting in a latent state within monocytes, granulocytes, B cells, and endothelial cells. Primary CMV infection or reactivation, however, can lead to serious clinical consequences, particularly in immunocompromised individuals. Symptoms of an active CMV infection may include fever, muscle and joint pain, and fatigue, though organ-specific dysfunctions such as retinitis, pneumonia, hepatitis, encephalitis, or colitis may also occur [9,11,12].

Diagnosing CMV infection is challenging. In routine clinical practice, molecular techniques and immunohistochemical (IHC) staining, which detect viral DNA or CMV antigens in tissue samples, are considered the gold standard (Appendix A) [4,13,14,15]. The latest European Crohn’s and Colitis Organisation (ECCO) guidelines from 2021 recommend CMV diagnostics in UC patients with active disease unresponsive to immunosuppressive treatment, particularly in steroid-resistant cases [4]. When CMV infection is confirmed, antiviral treatment is suggested to potentially reduce colectomy rates. However, evidence supporting this approach remains limited and conflicting.

Given the potential clinical significance of detecting concomitant CMV colitis in patients with UC exacerbations, we sought to evaluate the incidence of CMV infection among patients undergoing colectomy for drug-resistant UC. This study was a single-center, retrospective analysis conducted at a tertiary care facility during a period when CMV diagnostics were not routinely recommended or implemented. The primary objective was to assess the frequency of CMV infection in archival tissue samples and to compare the disease characteristics and surgical outcomes between patients with and without CMV infection.

## 2. Materials and Methods

### 2.1. Patients and Clinical Data

The study group comprised patients hospitalized in a tertiary referral center in Western Poland (University Clinical Hospital in Poznan) due to an exacerbation of UC that was unresponsive to conservative treatment, who were qualified for elective proctocolectomy. This retrospective study covered the period from 2009 to 2017. The exclusion criteria included a diagnosis of acute severe UC, the coexistence of gastrointestinal infections other than CMV, surgeries other than proctocolectomy, and the unavailability of high-quality postoperative tissue material.

Based on a review of medical records, selected factors were assessed, including demographics (age and gender), the course of UC (disease duration, number of hospitalizations and exacerbations, location of endoscopic lesions according to the Montreal classification, and endoscopic activity per the Mayo score), laboratory results (leukocyte and platelet counts, albumin levels, and C-reactive protein—CRP levels), and treatments administered (mesalazine—5-ASA, thiopurines, steroids, and biological therapy) [16,17]. Data on the postoperative course were also analyzed, such as complications (defined as postoperative wound complications, infections, bowel obstructions, and bleeding), postoperative mortality, the feasibility of restoring gastrointestinal continuity with the creation of an intestinal reservoir (J-pouch), the occurrence of pouchitis, and a complicated UC course after discharge from the hospital, defined as the need for rehospitalization within six months after surgery.

Additionally, follow-up data were collected to assess duration and survival outcomes. For this purpose, information on the one-year survival rate and overall follow-up duration was obtained from the hospital’s electronic records.

### 2.2. Histological and Immunohistochemical Assessments

For each case, we collected complete sets of formalin-fixed, paraffin-embedded (FFPE) blocks from surgical resections of the large intestine, as well as routine histological slides from these blocks (4 µm thick sections stained with hematoxylin and eosin [HE]), which were previously prepared for a standard histopathological diagnosis. The histological activity of inflammatory lesions was assessed using the Simplified Geboes Score, which evaluates variables such as abnormalities in the colonic tissue architecture, the presence of plasma cells, eosinophilic or neutrocyte infiltrates (in the mucosal lamina propria and epithelial layer), and epithelial layer damage (erosions and/or ulcerations) (Table 1) [18].

Additionally, for each case, HE-stained sections representing the area of most intense inflammatory changes were selected, and the corresponding FFPE block was identified. The selected FFPE blocks were then subjected to an additional IHC analysis. FFPE blocks were cut on a manual rotary microtome (AccuCut, Sakura—Sakura Finetek, Torrance, CA, USA) into 4 µm thick paraffin sections and mounted on extra-adhesive slides (SuperFrost Plus, MenzelGläser, Braunschweig, Germany). Sections were deparaffinized in xylene and alcohol and dried in an oven at 60 °C for 60 min. Epitope retrieval was performed using PT-Link solution at pH 9.0 for 20 min at 97 °C [DAKO Cat. No. K8000]. The sections were then cooled. For the staining procedure, commercially available primary monoclonal mouse antibodies against CMV (DAKO, CCH2 + DDG9, dilution 1:20) were used, along with the EnVision FLEX High pH visualization system and the Autostainer Link48 (DAKO) in accordance with the manufacturer’s recommendations. Sections were dehydrated in alcohol and xylene following staining.

An absolute number of CMV-positive cells was counted in each case showing positivity. The presence of ≥5 CMV-positive cells in a high-power field (HPF) served as the criterion for a positive result confirming CMV infection [16]. The density of CMV expression was further evaluated by calculating the mean number of CMV-positive cells per 1 mm^2^ of the tissue section. The microscopic evaluation was conducted by a specialist histopathologist experienced in the histopathological assessment of the gastrointestinal tract who was blinded to the clinical data of the study patients.

### 2.3. Statistical Analysis

The normality of the distribution of variables was tested using the Shapiro–Wilk test. To compare variables between the two groups, Student’s *t*-test, Welch’s test, or the Mann–Whitney test were employed. To examine the relationships between categorical variables, the Fisher exact test or the Fisher–Freeman–Halton test was used, following the verification of Cochran’s conditions. Relationships between continuous variables were examined using the Pearson linear correlation coefficient or Spearman’s rank correlation coefficient Rs. A *p*-value of <0.05 was considered statistically significant. Calculations were performed using Statistica 13 by TIBCO (Palo Alto, CA, USA) and PQStat by PQStat Software (version 1.8.6).

### 2.4. Bioethical Considerations

The protocol of the study was accepted by the Bioethics Committee of the Poznan University of Medical Sciences and the requirement for informed consent was waived due to the retrospective character of this study.

## 3. Results

**Figure 1:**
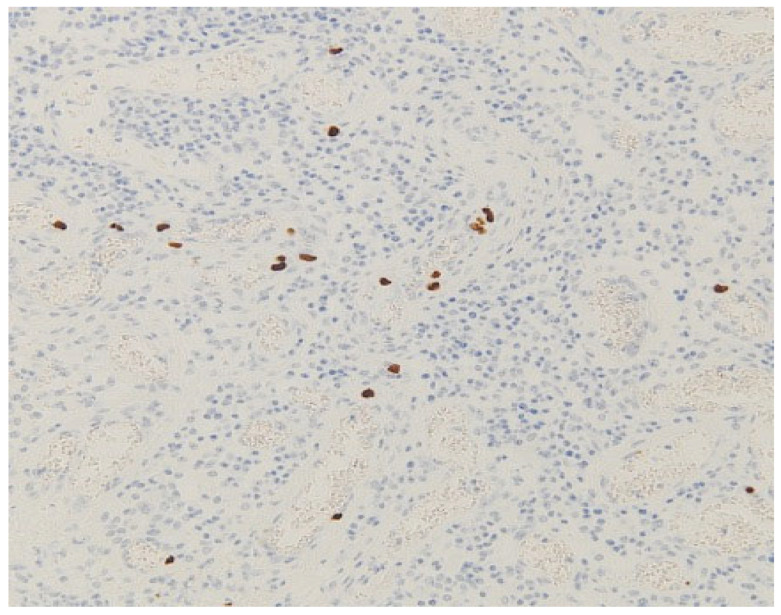
Immunohistochemical expression of cytomegalovirus in a surgical specimen from a patient with ulcerative colitis (magnification: 200×).

After applying the exclusion criteria, a total of 49 patients were assessed. CMV infection was identified in seven of these patients (14.3%) (Figure 1).

### 3.1. General Characteristics of the Study Groups

The two subgroups were comparable in terms of age and gender. Patients with a confirmed CMV infection at the time of surgery had a significantly shorter disease duration [0.4 years (IQR 0.08–1.5) vs. 5 years (IQR 2–14); *p* = 0.007] and higher CRP levels [76 (57) mg/L vs. 17 (73) mg/L, reference range: <5.0 mg/L; *p* = 0.02]. Additionally, they exhibited a trend toward higher leukocytosis and lower albumin concentrations, although these differences did not reach statistical significance. The steroid burden was numerically higher in CMV-positive patients, both in terms of steroid use at the time of surgery (71.4% vs. 45.2%) and the cumulative number of days of steroid use before colectomy [150 (445) vs. 90 (167) days]. However, these differences were also not statistically significant. The mean follow-up time in the whole cohort was 25 ± 24 months.

### 3.2. Postoperative Follow-Up Data After Colectomy

Postoperative mortality rates were higher among CMV-positive patients compared to CMV-negative patients (14.3% vs. 2.4%). The one-year survival rate was higher among patients without a CMV infection; however, the difference was not significant. Furthermore, CMV-positive patients were less likely to undergo subsequent surgical procedures, including stoma closure, pouch formation, and ileal–pouch anastomosis. A detailed comparative analysis of CMV-positive and CMV-negative patients is presented in Table 2.

### 3.3. Histopathological Data

Histopathological activity, assessed using the Simplified Geboes Score, did not differ significantly between the two subgroups, both in terms of the overall score [9 (min–max: 3–12) vs. 10 (min–max: 7–12) for CMV-negative and CMV-positive specimens, respectively] and when comparing the individual components of the score, no statistically significant differences were observed. The mean density of CMV-positive cells in colonic tissue was 1.04 ± 1.05 cells/mm^2^. No significant correlations were observed between the density of CMV expression and UC histopathological activity as measured by the Simplified Geboes Score, either for the overall score or for its individual components. Similarly, no statistical associations were found between the CMV tissue density and levels of CRP, leukocytosis, albumin levels, or platelet count.

## 4. Discussion

Our findings demonstrate that CMV colonic infection is a clinical concern among patients with active ulcerative colitis (UC) refractory to drug therapy, particularly those exhibiting steroid resistance.

In our study cohort, the incidence of CMV infection was clinically relevant, as it was seen in 14% of patients. This is consistent with findings reported in the literature, where the frequency of CMV colitis among UC patients with active, treatment-resistant disease ranges from 2% to 38%, depending on diagnostic criteria and study design [19,20,21,22,23,24]. Discrepancies in these figures may result from differences in diagnostic criteria across studies (Appendix A) and patients’ characteristics. Although our analysis focused on patients with refractory UC undergoing colectomy, CMV colitis may also occur in patients with conventionally progressing UC, including those without documented steroid-refractory disease. Recent studies indicate that CMV reactivation may be present even in hospitalized IBD patients who do not meet classical high-risk criteria, potentially influencing clinical outcomes such as prolonged hospitalization and the need for treatment escalation [25,26]. This highlights the importance of a broader diagnostic approach, including histopathological and molecular testing, especially in patients with a severe inflammatory burden, regardless of steroid responsiveness.

The initial CMV diagnosis often involves serological testing. IgM antibodies suggest a recent or acute infection, while IgG antibodies suggest a past exposure [27,28]. However, negative results for both IgG and IgM do not exclude CMV-related inflammation and must be interpreted with caution. A CMV carrier status implies a latent infection without active viral replication, while active replication can cause tissue damage and clinical symptoms indicative of a CMV infection. That is why, to distinguish between these states, the presence of high viral tissue loads should be confirmed [16,17,27,29].

PCR-based detection of CMV appears to be a reliable method. Studies show that CMV-DNA loads exceeding 250 copies/mg of tissue are associated with an active infection [17,29,30,31]. This test exhibits high sensitivity (65–100%) and specificity (42–92%), depending on the type of tissue examined [27]. Costs and the relatively limited routine availability of molecular CMV diagnostics are potential limitations of PCR testing. Alternatively, a histopathological examination of colonic specimens with subsequent IHC detection of CMV antigens can confirm significant CMV replication [16,32,33,34]. According to Zagórowicz et al., ≥5 CMV-positive cells per section are associated with a higher risk of colectomy among UC patients [16]. Similarly, Jones et al. proposed this threshold as a potential guide for antiviral treatment decisions for steroid-refractory UC [32].

The role of antiviral therapy in patients with UC and signs of an active CMV infection remains controversial. Some studies suggest that ganciclovir can reduce the colectomy risk and improves short-term outcomes in steroid-refractory patients [30,31,32]. For example, Robin et al. reported a clinical benefit from antiviral treatment in patients with a high CMV tissue load and poor immunosuppressive response [31]. Conversely, a recent study by Huang et al. (2024) found no benefit in patients with acute severe UC, despite a high prevalence of CMV positivity [35]. These conflicting results underscore the need for well-designed prospective studies to identify which subgroups may benefit from antiviral intervention.

An important clinical question is which UC patients should be tested for CMV infection. An analysis of the literature identifies significant risk factors, including older age, comorbidities, severe disease course, elevated inflammatory marker levels, and immunosuppressive therapies, particularly prolonged steroid use [20,24,36,37,38]. In our cohort, no significant differences were observed in leukocyte and platelet counts or albumin levels, nor in endoscopic and histological activity, suggesting that these factors lack utility in predicting a higher probability of subsequent CMV colitis. On the other hand, CMV infection was more frequently observed in those with a more fulminant disease course, defined by a shorter disease duration with a significant CRP elevation. CMV-positive patients were also more frequently exposed to steroids, both in terms of usage frequency and cumulative treatment duration. This suggests that patients with a dynamic, aggressive disease course are more susceptible to CMV reactivation, possibly due to severe inflammation. Conversely, the dynamic and aggressive disease progression among these patients may result from overlapping active UC and CMV colitis.

To the best of our knowledge, our study is the first to assess the possible association of preoperative CMV infection with short- and long-term prognoses after surgery. We found that CMV-positive patients were at increased risk of a lower probability of gastrointestinal continuity restoration. Numerically, CMV colitis was also associated with an increased risk of pouchitis and higher postoperative mortality.

Our study has several limitations. One of the most significant is the relatively small sample size. However, after analyzing the existing medical data in this area, it should be emphasized that this is still one of the largest cohorts studied to date. Moreover, since CMV infection was identified only postoperatively, we were unable to assess whether earlier detection and antiviral treatment might have influenced the prognosis or reduced the need for surgery. Nevertheless, our primary aim was to evaluate the frequency of CMV colitis and its potential clinical implications during a period when such assessment was not routinely performed. It is important to emphasize that the absence of preoperative CMV screening in our study reflects historical diagnostic practices rather than current standards. This context should be considered when comparing our findings with more recent data. Furthermore, our study highlights how rapidly evolving knowledge in recent years has significantly influenced clinical practice, potentially leading to improved care for patients with UC.

Another potential limitation is the retrospective nature of the study. However, considering the decreasing number of colectomies in UC in the era of innovative treatments, conducting a prospective study would be extremely time-consuming.

## 5. Conclusions

Our findings show that CMV colitis is not an uncommon condition and should be considered, particularly among UC patients with a dynamic, aggressive disease course unresponsive to standard treatment, especially steroids. Proper identification of CMV infection can increase the frequency of antiviral treatment, hypothetically decreasing the need for colectomy in selected patients. This is especially important since the prognosis following colectomy appears poorer for patients with concomitant CMV colitis at the time of surgery. However, further prospective, multicenter research is essential to optimize diagnostic and therapeutic strategies for this patient population.

## Figures and Tables

**Table 1:** The Simplified Geboes Score, assessing the histological activity of ulcerative colitis [18].

Grade 0: No inflammatory activity	0.0 No abnormalities 0.1 Presence of architectural changes 0.2 Presence of architectural changes and a chronic mononuclear cell infiltrate
Grade 1: Basal plasma cells	1.0 No increase 1.1 Mild increase 1.2 Marked increase
Grade 2 A: Eosinophils in the lamina propria	2A.0 No increase 2A.1 Mild increase 2A.2 Marked increase
Grade 2 B: Neutrophils in the lamina propria	2B.0 No increase 2B.1 Mild increase 2B.2 Marked increase
Grade 3: Neutrophils in the epithelium	3.0 None 3.1 <50% of crypts involved 3.2 >50% of crypts involved
Grade 4: Epithelial injury [in crypts and surface epithelium]	4.0 None 4.1 Marked attenuation 4.2 Probable crypt destruction: probable erosions 4.3 Unequivocal crypt destruction: unequivocal erosion 4.4 Ulcer or granulation tissue

**Table 2:** Comparison of the clinical presentation of patients with ulcerative colitis with and without concomitant cytomegalovirus (CMV) infection at the time of surgery.

Clinical Characteristics	CMV Positive	CMV Negative	*p*-Value
n (%)	7 (14.28%)	42 (85.71%)	
Age [median (IQR)], years	37 (24–55)	45 (28–59)	0.60
Sex [female], n (%)	2 (28.57%)	16 (38.10%)	1.00
Age at diagnosis, [median (IQR)], years	37 (22–55)	29 (22–49)	0.50
Duration of UC, [median (IQR)], years	0.4 (0.08–1.5)	5 (2–14)	0.007
Number of hospitalizations before surgery, [median (min–max)]	2 (1–6)	3 (1–8)	0.47
Number of exacerbations before hospitalization, [median (min–max)]	2 (0–4)	3 (1–10)	0.09
Laboratory data:			
C-reactive protein [mg/L]; the reference range is below 5 mg/L, (SD)	76 (57)	17 (73)	0.02
Total WBC count [×1000/µL]; the reference range is between 3.90 and 11.00 × 1000/µL, (SD)	15 (6)	8 (5)	0.12
Platelet count [×1000/µL]; the reference range is between 130 and 400 × 1000/µL, (SD)	289 (114)	332 (121)	0.52
Albumin [g/dL]; the reference range is between 3.50 and 5.20 g/dL, (SD)	2.7 (1.1)	3.4 (0.7)	0.15
Extent of UC:			1.00
E2 Left-sided colitis, n (%)	3 (43%)	19 (45%)	
E3 Extensive colitis, n (%)	4 (57%)	23 (55%)	
Endoscopic Mayo Score:			1.00
Mayo 2, n (%)	0 (0%)	1 (2%)	
Mayo 3, n (%)	7 (100%)	41 (98%)	
Steroid use at the time of surgery, n (%)	5 (71%)	19 (45%)	0.41
Duration of steroid therapy before surgery [number of days] (SD)	150 (446)	90 (167)	0.41
Treatment:			
5-ASA, n (%)	6 (86%)	33 (79%)	1.00
Thiopurines, n (%)	1 (14%)	10 (24%)	1.00
Infliximab therapy, n (%)	2 (29%)	3 (7%)	0.15
Smokers, n (%)	1 (14%)	2 (5%)	0.39
Presence of comorbidities, n (%)	4 (57%)	26 (62%)	1.00
Postoperative complications, n (%)	4 (57%)	18 (43%)	0.68
Death after surgery, n (%)	1 (14%)	1 (2%)	0.26
Complicated course of UC after discharge from the hospital, n (%)	3 (43%)	18 (43%)	1.00
Restoration of gastrointestinal continuity, n (%)	1 (14%)	30 (71%)	0.001
Pouchitis, n (%)	1 (100%)	8 (27%)	0.29
Simplified Geboes Score for UC: Severity of histopathological changes [median (min-max)]	10 (7–12)	9 (3–12)	0.5
Follow-up duration, Months [mean (SD)]	31 (28)	18 (17)	0.2
One-year survival rate, n (%)	6/7 (85.7%)	41/42 (97.6%)	0.152

Abbreviations: 5-ASA, 5-aminosalicylic acid; CMV, cytomegalovirus; IQR, interquartile range; SD, standard deviation; UC, ulcerative colitis; WBC, white blood count. SI conversion factors: To convert C-reactive protein (CRP) levels to mg/L, multiply by 1.0; to convert the WBC count to ×10^9^/L, multiply by 10^6^; to convert the platelet count to ×10^9^/L, multiply by 10^6^; albumin levels are g/dL, multiply the value by 10 to obtain the concentration in g/L.

## Data Availability

The data are available upon reasonable request.

## Abbreviations

The following abbreviations are used in this manuscript:

CMV	Cytomegalovirus
UC	Ulcerative colitis
IHC	Immunohistochemistry
FFPE	Formalin-fixed, paraffin-embedded
HE	Hematoxylin and eosin
CRP	C -reactive protein
WBC	White blood cell
HPF	High-power field
5-ASA	5-Aminosalicylic acid
IQR	Interquartile range
SD	Standard deviation
ECCO	European Crohn’s and Colitis Organisation
PCR	Polymerase chain reaction

## Appendix A

**Table A1:** Diagnostic methods used for the CMV evaluation in patients with inflammatory bowel disease [17,27,28,30,31].

Type of Method	Interpretation	Advantages	Limitations
Serological tests: CMV IgM antibodies CMV IgG antibodies	- Indicate a past or recent CMV infection, but may persist for an extended period; - Typically appear within 2–4 weeks after infection; - The presence of IgG indicates a previous exposure to CMV; - Appear at approximately 2–4 weeks postinfection.	- Widely available, low cost; - Simple blood test; - Reliable in immunocompetent individuals.	- The persistence of IgM after an acute infection may lead to misinterpretation; - Unreliable in immunosuppressed patients, who may not produce detectable antibodies; - Does not localize infection (only suggests systemic presence).
PCR of blood	- A rapid and sensitive method, but not always sufficient alone.	- Detects low levels of viral DNA in peripheral blood; - Enables the early detection of infection; - Results available within 24–48 h; - High sensitivity, suitable for monitoring the antiviral treatment response.	- A negative PCR result does not exclude intestinal infection, as CMV may replicate locally in the bowel wall without systemic viremia; - Does not localize the site of infection, only indicates systemic presence (may be negative in those with a local disease); - The interpretation must consider the clinical context, as low-level viremia may be clinically insignificant.
IHC and PCR for intestinal tissue histopathology	Gold standard for diagnosis—allows the direct detection of CMV at the site of inflammation (intestinal wall).	- The presence of CMV in intestinal cells confirms an active infection; - Enables an assessment of the number and distribution of infected cells; - Allows an evaluation of inflammatory activity and tissue damage from the same specimen; - Supports therapeutic decisions, such as initiating antiviral treatment.	- Requires colonoscopy with the collection of tissue samples for a histopathological examination; - Evaluates viral presence in the sampled tissue only—does not detect systemic infection; - Insufficient sampling or absence of infected areas may yield false-negative results; - Not all medical centers routinely perform this test; - Requires interpretation by an experienced gastrointestinal pathologist.
Histopathology		- Direct visualization and characteristic enlarged cells with ‘’owl’s eye” intranuclear inclusions; - Confirms the presence of CMV in specific tissues; - Routine availability, can be performed on formalin-fixed, paraffin-embedded tissue from a standard biopsy; - Compatibility with immunohistochemistry.	- Low sensitivity to mild infections or absent in early disease; - Requires an experienced pathologist; - Delay in diagnosis (several days); - Biopsies may miss infected areas; - Cannot distinguish between a latent and active infection (clinical context or additional testing—PCR).

## References

[1] Ungaro R., Mehandru S., Allen P.B., Peyrin-Biroulet L., Colombel J.F. (2017). Ulcerative colitis. Lancet.

[2] Raine T., Bonovas S., Burisch J., Kucharzik T., Adamina M., Annese V., Bachmann O., Bettenworth D., Chaparro M., Czuber-Dochan W. (2022). ECCO guidelines on therapeutics in ulcerative colitis: Medical treatment. J. Crohn’s Colitis.

[3] Rubin D.T., Ananthakrishnan A.N., Siegel C.A., Sauer B.G., Long M.D. (2019). ACG clinical guideline: Ulcerative colitis in adults. Am. J. Gastroenterol..

[4] Kucharzik T., Ellul P., Greuter T., Rahier J.F., Verstockt B., Abreu C., Albuquerque A., Allocca M., Esteve M., Farraye F.A. (2021). ECCO guidelines on the prevention, diagnosis, and management of Infections in inflammatory bowel disease. J. Crohn’s Colitis.

[5] Criscuoli V., Casà A., Orlando A., Pecoraro G., Oliva L., Traina M., Rizzo A., Cottone M. (2004). Severe acute colitis associated with CMV: A prevalence study. Dig. Liver Dis..

[6] Kojima T., Watanabe T., Hata K., Shinozaki M., Yokoyama T., Nagawa H. (2006). Cytomegalovirus infection in ulcerative colitis. Scand. J. Gastroenterol..

[7] Maconi G., Colombo E., Zerbi P., Sampietro G.M., Fociani P., Bosani M., Cassinotti A., Casini V., Russo A., Ardizzone S. (2005). Prevalence, detection rate and outcome of cytomegalovirus infection in ulcerative colitis patients requiring colonic resection. Dig. Liver Dis..

[8] Griffiths P., Reeves M. (2021). Pathogenesis of human cytomegalovirus in the immunocompromised host. Nat. Rev. Microbiol..

[9] Gugliesi F., Pasquero S., Griffante G., Scutera S., Albano C., Pacheco S.F.C., Riva G., Dell’Oste V., Biolatti M. (2021). Human cytomegalovirus and autoimmune diseases: Where are we?. Viruses.

[10] Yokoyama Y., Yamakawa T., Hirano T., Kazama T., Hirayama D., Wagatsuma K., Nakase H. (2020). Current diagnostic and therapeutic approaches to cytomegalovirus infections in ulcerative colitis patients based on clinical and basic research data. Int. J. Mol. Sci..

[11] Griffiths P., Baraniak I., Reeves M. (2015). The pathogenesis of human cytomegalovirus. J. Pathol..

[12] Rowshani A.T., Bemelman F.J., van Leeuwen E.M., van Lier R.A., ten Berge I.J. (2005). Clinical and immunologic aspects of cytomegalovirus infection in solid organ transplant recipients. Transplantation.

[13] Maresca R., Varca S., Di Vincenzo F., Ainora M.E., Mignini I., Papa A., Scaldaferri F., Gasbarrini A., Giustiniani M.C., Zocco M.A. (2024). Cytomegalovirus infection: An underrated target in inflammatory bowel disease treatment. J. Clin. Med..

[14] Zidar N., Ferkolj I., Tepeš K., Štabuc B., Kojc N., Uršič T., Petrovec M. (2015). Diagnosing cytomegalovirus in patients with inflammatory bowel disease-by immunohistochemistry or polymerase chain reaction?. Virchows Arch..

[15] Goodman A.L., Murray C.D., Watkins J., Griffiths P.D., Webster D.P. (2015). CMV in the gut: A critical review of CMV detection in the immunocompetent host with colitis. Eur. J. Clin. Microbiol. Infect. Dis..

[16] Zagórowicz E., Bugajski M., Wieszczy P., Pietrzak A., Magdziak A., Mróz A. (2016). Cytomegalovirus infection in ulcerative colitis is related to severe inflammation and a high count of cytomegalovirus-positive cells in biopsy is a risk factor for colectomy. J. Crohns Colitis..

[17] Eder P., Łodyga M., Gawron-Kiszka M., Dobrowolska A., Gonciarz M., Hartleb M., Kłopocka M., Małecka-Wojciesko E., Radwan P., Reguła J. (2023). Guidelines for the management of ulcerative colitis. Recommendations of the Polish Society of Gastroenterology and the Polish National Consultant in Gastroenterology. Prz. Gastroenterol..

[18] Jauregui-Amezaga A., Geerits A., Das Y., Lemmens B., Sagaert X., Bessissow T., Lobatón T., Ferrante M., Van Assche G., Bisschops R. (2017). A Simplified Geboes score for ulcerative colitis. J. Crohn’s Colitis.

[19] Meeralam Y., Al Qurashi B., Al Masoudi A., Alhejaili T.L., Khayat M., Aljoaid A.M., Al Harthi W., Hafiz W.A., Shariff M.K. (2023). Cytomegalovirus colitis in a patient with ulcerative colitis on vedolizumab monotherapy. Cureus.

[20] Lee H.S., Park S.H., Kim S.H., Kim J., Choi J., Lee H.J., Kim W.S., Lee J.M., Kwak M.S., Hwang S.W. (2016). Risk factors and clinical outcomes associated with cytomegalovirus colitis in patients with acute severe ulcerative colitis. Inflamm. Bowel Dis..

[21] Maher M.M., Nassar M.I. (2009). Acute cytomegalovirus infection is a risk factor in refractory and complicated inflammatory bowel disease. Dig. Dis. Sci..

[22] Gauss A., Rosenstiel S., Schnitzler P., Hinz U., Rehlen T., Kadmon M., Ehehalt R., Stremmel W., Zawierucha A. (2015). Intestinal cytomegalovirus infection in patients hospitalized for exacerbation of inflammatory bowel disease: A 10-year tertiary referral center experience. Eur. J. Gastroenterol. Hepatol..

[23] Dimitroulia E., Spanakis N., Konstantinidou A.E., Legakis N.J., Tsakris A. (2006). Frequent detection of cytomegalovirus in the intestine of patients with inflammatory bowel disease. Inflamm. Bowel Dis..

[24] Kambham N., Vij R., Cartwright C.A., Longacre T. (2004). Cytomegalovirus infection in steroid-refractory ulcerative colitis: A case-control study. Am. J. Surg. Pathol..

[25] Weng M.T., Tung C.C., Lee Y.S., Leong Y.L., Shieh M.J., Shun C.T., Wang C.Y., Wong J.M., Wei S.C. (2017). Cytomegalovirus colitis in hospitalized inflammatory bowel disease patients in Taiwan: A referral center study. BMC Gastroenterol..

[26] Domènech E., Vega R., Ojanguren I., Hernández A., Garcia-Planella E., Bernal I., Rosinach M., Boix J., Cabré E., Gassull M.A. (2008). Cytomegalovirus infection in ulcerative colitis: A prospective, comparative study on prevalence and diagnostic strategy. Inflamm. Bowel Dis..

[27] Beswick L., Ye B., van Langenberg D.R. (2016). Toward an algorithm for the diagnosis and management of CMV in patients with colitis. Inflamm. Bowel Dis..

[28] Zagórowicz E., Przybysz A., Szlak J., Magdziak A., Wieszczy P., Mróz A. (2018). Detection of cytomegalovirus by immunohistochemistry of colonic biopsies and quantitative blood polymerase chain reaction: Evaluation of agreement in ulcerative colitis. Scand. J. Gastroenterol..

[29] Kim J.W., Boo S.J., Ye B.D., Kim C.L., Yang S.K., Kim J., Kim S.A., Park S.H., Park S.K., Yang D.H. (2014). Clinical utility of cytomegalovirus antigenaemia assay and blood cytomegalovirus DNA PCR for cytomegaloviral colitis patients with moderate to sSevere ulcerative colitis. J. Crohn’s Colitis.

[30] Pillet S., Pozzetto B., Roblin X. (2016). Cytomegalovirus and ulcerative colitis: Place of antiviral therapy. World J. Gastroenterol..

[31] Roblin X., Pillet S., Oussalah A., Berthelot P., Del Tedesco E., Phelip J.M., Chambonnière M.L., Garraud O., Peyrin-Biroulet L., Pozzetto B. (2011). Cytomegalovirus load in inflamed intestinal tissue is predictive of resistance to immunosuppressive therapy in ulcerative colitis. Am. J. Gastroenterol..

[32] Jones A., McCurdy J.D., Loftus E.V., Bruining D.H., Enders F.T., Killian J.M., Smyrk T.C. (2015). Effects of antiviral therapy for patients with inflammatory bowel disease and a positive intestinal biopsy for cytomegalovirus. Clin. Gastroenterol. Hepatol..

[33] Kandiel A., Lashner B. (2006). Cytomegalovirus colitis complicating inflammatory bowel disease. Am. J. Gastroenterol..

[34] Juric-Sekhar G., Upton M.P., Swanson P.E., Westerhoff M. (2017). Cytomegalovirus (CMV) in gastrointestinal mucosal biopsies: Should a pathologist perform CMV immunohistochemistry if the clinician requests it?. Hum. Pathol..

[35] Huang D., Rennie M., Krasovec A., Nagubandi S., Liu S., Ge E., Khehra B., Au M., Sivagnanam S., Kwan V. (2024). Impact of cytomegalovirus on outcomes in acute severe ulcerative colitis: A retrospective observational study. Ther. Adv. Chronic Dis..

[36] Qin Y., Wang G., Kong D., Li G., Wang H., Qin H. (2021). Risk factors of cytomegalovirus reactivation in ulcerative colitis patients: A meta-analysis. Diagnostics.

[37] Henmi Y., Kakimoto K., Inoue T., Nakazawa K., Kubota M., Hara A., Mikami T., Naka Y., Hirata Y., Hirata Y. (2018). Cytomegalovirus infection in ulcerative colitis assessed by quantitative polymerase chain reaction: Risk factors and effects of immunosuppressants. J. Clin. Biochem. Nutr..

[38] Olaisen M., Rydning A., Martinsen T.C., Nordrum I.S., Mjønes P., Fossmark R. (2014). Cytomegalovirus infection and postoperative complications in patients with ulcerative colitis undergoing colectomy. Scand. J. Gastroenterol..

